# Prevalence of low birth weight and its associated factors: Hospital based cross sectional study in Nepal

**DOI:** 10.1371/journal.pgph.0001220

**Published:** 2022-11-02

**Authors:** Pratibha Thapa, Amod Poudyal, Rajan Poudel, Dipak Prasad Upadhyaya, Ashish Timalsina, Rama Bhandari, Jijeebisha Baral, Rabindra Bhandari, Prakash Chandra Joshi, Pratiksha Thapa, Nabin Adhikari

**Affiliations:** 1 Central Department of Public Health, Institute of Medicine, Tribhuvan University, Kathmandu, Nepal; 2 School of Medicine, Case Western Reserve University, Cleveland, OH, United States of America; 3 James P. Grant School of Public Health, BRAC University, Dhaka, Bangladesh; 4 Ace Institute of Management, Pokhara University, Kathmandu, Nepal; 5 Research and Development Department, Dhulikhel Hospital, Dhulikhel, Kavre, Nepal; University of Adelaide, AUSTRALIA

## Abstract

Low birth weight is still an important public health problem worldwide. It is a major contributor to neonatal death in developing countries, including Nepal. The government of Nepal has developed and implemented different programs to improve maternal and neonatal health, including baby’s birth weight. However, low birth weight is a major maternal and child health challenge. Maternal factors determining the birth weight of neonates have been poorly assessed in previous studies in Nepal. Thus, this study aims to assess the prevalence and risk factors associated with low birth weight in Nepal. An institution-based descriptive cross-sectional study was carried out in Paropakar Maternity Hospital and Tribhuvan University Teaching Hospital of Kathmandu district among 308 postnatal mothers. The data was collected through the face-to-face interview technique. The data was entered in EpiData 3.1 and exported to Statistical Package and Service Solutions version 21 for analysis. Multivariate logistic regression was used to obtain an adjusted odds ratio, while p-value < 0.05 with 95% Confidence Interval (CI) was considered significant. The findings showed that 15.3% of the children had low birth weight. The mean and standard deviation of childbirth weight was 2.96±0.59 kg. Mothers belonged to Dalit ethnic (AOR = 2.9, 95% CI = 1.2–7.1), Antenatal Care visited three or fewer (AOR = 2.6, 95%CI = 1.0–6.6) and did not comply with Iron and Folic Acid supplementation (AOR = 2.1, 95% CI = 1.0–4.4) were significantly associated with low birth weight. Nearly one in every six children had low birth weight. Maternal health services such as antenatal care and compliance with a recommended dose of maternal micronutrients significantly impact on birth weight. Maternal and neonatal health programs should consider these factors to reduce adverse birth outcomes in Nepal.

## Introduction

Birth weight has been one of the predictors of child mortality and morbidity. According to the World Health Organization (WHO), Low birth weight (LBW) live-born infants with a birth weight of less than 2500 grams, irrespective of the gestational age [1]. Epidemiological observations reflect that LBW babies have 20 times higher odds of dying than heavier infants [2]. Globally, LBW contributes to 40–60% of newborn mortality [3]. The baby’s birth weight is a sensitive indicator of the overall health situation of the population. LBW babies are at risk of cognitive deficits, motor delays, cerebral palsy, and other behavior and psychological problems [4, 5]. Furthermore, it is an indirect indicator of maternal health and a predictive indicator of potential neonatal death, the child’s malnutrition and cardiovascular disease risks in later life [6].

The global prevalence of low birth weight is estimated to be 15% to 20% of all births representing over 20 million births in a year [7]. There is wide variation in the prevalence of low birth weight across regions; however, evidence shows the nearly half of low births weight occurs in low and middle-income countries and especially in the most vulnerable population [8]. The regional estimates of low birth weight show 28% in south Asia, 6% east Asia and the Pacific, 13% in sub-Saharan Africa and 9% in Latin America [9]. According to Nepal Demographic Health Survey 2016, the prevalence of low birth weight in Nepal was 12%, which has stagnant since 2011 [10]. Similarly, most hospital-based cross-sectional studies conducted in Nepal show the prevalence of LBW varies from 12% to 39.6% [11–14].

Evidence suggests that factors such as iron folic acid intake [15], maternal weight gain during pregnancy [16], preterm birth [17], mothers having inadequate antenatal care visits [18], anemic mothers [19] and smoking habits [20] were associated with low birth weight. Previous studies conducted in Nepal have shown that maternal education, maternal weight, maternal height, maternal age and lack of consumption of nutritious food during pregnancy were significantly associated with low birth weight [16, 21, 22]. The government of Nepal has recognized nutrition as a priority program of Nepal. Nepal aims to reduce LBW to less than 1.4% by 2030 [23]. To reduce low birth weight government has taken different strategies such as counseling during ANC visits, routine supplementation of IFA to pregnant women, providing nutritious food such as super cereals to pregnant women residing in highly food insecure areas and awareness of early pregnancy, smoking and alcohol during pregnancy [23]. Despite these efforts, the prevalence of low birth weight remains a major public health problem in Nepal [10].

Various factors were explored to identify the cause of low birth weight in different countries [16, 24, 25]. The majority of these studies focused on sociodemographic, cultural and nutritional factors. However, other factors such as ANC visit, consumption of IFA, maternal knowledge and tobacco/alcohol consumption have not been well studied. Hence this study would help to better understand the associated risk factors of low birth weight of babies in our context. This study’s finding also help generate local evidence for informed planning of the intervention to address the problem of low birth weight.

## Methods

### Study design

A cross-sectional study was conducted among two tertiary hospitals from September 2019 to August 2020 by using quantitative methods.

### Study population

Our study population was postnatal mothers. We included mothers who had singleton pregnancy and recently delivered liv- birth baby admitted in the postnatal ward of Tribhuvan University Teaching Hospital (TUTH) and Paropakar Maternity and Women’s Hospital (PMWH). We excluded the mothers with any serious obstetric or medical conditions, mothers who did not have ANC cards, who had multiple pregnancies, whose last menstrual period was not exactly known, who had a history of some complications like antepartum hemorrhage and neonates with congenital malformations, had medical conditions like Diabetes, Hypertension, cardiac diseases or chronic infections and who required constant medical support and monitoring.

### Study site

The study sites were Tribhuvan University Teaching Hospital (TUTH) and Paropakar Maternity and Women’s Hospital (PMWH) of Kathmandu district, Nepal. Kathmandu district is located in Kathmandu Valley, Bagmati Province of Nepal. It covers an area of 413.69 km^2^ (153 sq mi), and is the most densely populated district. It’s headquarter is Kathmandu Metropolitan City [26]. Kathmandu district has many tertiary hospitals, which is the catchment area for most people from several regions of Nepal. TUTH and PMWH are both public tertiary health centers as well as referral centers. These hospitals have a substantial caseload, with PMWH being the main public maternity hospital in Kathmandu and TUTH being a University hospital providing all kinds of maternity services to a large group of women inside and outside Kathmandu valley. People attending these hospitals have diverse ethnicity, culture and lifestyle, so that more information can be obtained.

### Sample size and sampling method

A total of 308 mothers were interviewed with equal samples (154) taken from each hospital which was calculated using the formula n = z^2^pq/d^2^, considering the prevalence of low birth weight as 23.6% from the previous study conducted at Bharatpur Nepal [27], at 95% CI, 5% tolerable error and 10% non-response rate. Two tertiary level hospitals were selected purposively. Equal samples were taken from each hospital. The list of mothers was obtained from the postnatal wards of the respective hospital. The number of beds occupied was obtained from a ward in charge and the number was almost similar (TUTH 312 vs PMWH 304). Samples were selected through a systematic random sampling method in each hospital. The random number was generated, and the research participants were sorted in ascending bed numbers. The first participant’s bed number was selected by lottery method. Then, each study participant was selected by using systematic random sampling. The sampling interval(K) was 2. Data was collected on the day of discharge of participants before leaving the facility.

### Data collection tools and techniques

We conducted face-to-face interviews among postnatal mothers using a semi-structured questionnaire. We also used the ANC card and Maternity register to confirm the information. The tools were developed based on study objectives through extensive literature review and pretested among 10% of non- sampled population in postnatal ward of TU Teaching hospital. PT, NA and two trained enumerators were involved in data collection from February 20, 2020 to April 7, 2020.

### Dependent variables

Birth weight was reported in grams which was operationalized as a continuous variable. We constructed a binary variable for low birth weight based on the weight of birth <2500 gm as defined by WHO [28].

### Independent variables

Independent variables were selected based on previously published studies [23, 27, 29–31]. Independent variables were broadly classified into socio-demographic factors, maternal factors, health service-related factors, maternal knowledge and compliance to iron and folic acid supplementation.

Sociodemographic factors included age of postnatal mother (categorized into 15 to 19, 20 to 24, 25 to 29, 30 to 34, 35 and above), ethnicity (categorized into Dalit and non-Dalit), education of mother (categorized into illiterate, informal, primary, secondary and higher education), occupation (categorized into agriculture, service, business and housewife). Maternal obstetric factors included the height of mother (categorized into less than 145 cm and more than 145 cm), weight gain during pregnancy (categorized into less than 10 kg, 10 kg or more), gestational age at birth (categorized into less than 37 weeks, 37 or more weeks), parity (categorized into primiparous, multiparous and grand multiparous), birth interval (categorized into less than 2 years and more than 2 years), pregnancy intention (categorized into unintended and intended), history of abortion Behavioral factors included tobacco and alcohol use. Diet-related factors included dietary patterns, food taboos during pregnancy. Health service-related factors included ANC visit (categorized into less than 4 visits and more than or 4 visits), intake of deworming (categorized into Yes and No), hemoglobin level recorded at first ANC visit (categorized into less than 11 gm/dl and 11gm/dl or more). Maternal compliance with compliance to iron-folic acid was also assessed. Compliance was defined as the uptake of IFA supplementation by pregnant women daily. The mother was determined to have compliance if she uptakes at least 80% of the recommended dose (categorized into non-compliance if uptake less than 144 tablets and compliance if uptake more than or equal to 144 tablets) [32, 33]. Maternal knowledge of anemia and IFAS variables was categorized into adequate and inadequate based on the mean score.

### Statistical analysis

Data was entered in EpiData version 3.1. After confirming the completeness, data were exported to IBM SPSS Version 21 for further analysis. Univariate analysis was conducted using frequencies, percentages, mean and standard deviation. Multi-collinearity was assessed before logistic regression using the variance inflation factor (VIF). The decision criterion for excluding of variable from the regression model was set out as those with VIF values greater than 10. The goodness of fit the regression model was tested by applying the of Hosmer and Lemeshow chi-square test. Those variables significantly associated in the univariate analysis at 95% confidence level, p-value less than 0.05 were included in the multivariate model. We applied Multivariate logistic regression analysis adjusting for covariates such as ethnicity, IFA compliance, and ANC visit to identify the factors associated with low birth weight. The unadjusted and adjusted odds ratio with 95% confidence intervals were reported. P-value less than 0.05 was considered statistically significant.

### Ethics statement

The ethical review committee approved the study at the Institute of Medicine (approval no: 258(6–11) E2 076/077) and Nepal Health Research Council (approval no: 22/2020 MT). Permission was taken from both hospitals. Participants provided verbal and written consent to participate in the study, which was voluntary and anonymous.

## Results

### Distribution of participants by socio-demographic factors, maternal health service and birth weight-related factors

Table 1 describes the sociodemographic characteristics of the research participants. The majority of the participants belonged to age group 20–24 years of age (35.7%). The mean age of the respondent was 25.7 ± 4.8. The response rate in this study was 100%. Majority of the participants (43.5%) were Brahmin/Chhetri. Almost half of the participants (46.1%) had a higher educational level. More than three-fourths (77.6%) of the participants were homemakers. The majority (96.1%) of the participants had a height of more than and equal to 145cm. About two-third (61.7%) of the respondents had the weight gain of at least 10 kg during the pregnancy. The average weight gain during the pregnancy was 11.04 ± 4.15kg. Majority of the participants (90.6%) had more than 37 weeks of gestation. Majority (91.6%) of participants had attended four or more ANC visits during their pregnancy. More than half (58.4%) of the respondents did not consume deworming tablet during pregnancy. More than four-fifths (87.7%) of the respondents were non-anemic compared to 12.3% who were anemic. The mean hemoglobin level recorded at first ANC visits was 12.27 ± 1.28 gm/dl. Majority (97.4%) of participants consumed IFA tablets during pregnancy. More than three-fourths (77.6%) of the respondents had compliance to IFA supplementation. Most of the respondents (84.7%) had given birth to at least 2.5 kg of baby, while 15.3% had given birth to less than 2.5 kg of a baby. The mean birth weight was 2.96 ±0.59 kg.

**Table 1:** Distribution of participants by sociodemographic factors, maternal health service and birth weight-related factors.

Sociodemographic	Frequency	Percentage
**Age (in years)**		
<20 years	24	7.7
20–29 years	217	70.4
≥30 years	67	21.9
**Ethnicity**		
Dalit	44	14.3
Non Dalit	264	85.7
**Educational status**		
Illiterate	11	3.6
Informal education	16	5.2
Formal	281	91.2
**Occupational status**		
Employed	69	22.4
Homemaker	239	77.6
**Weeks of gestation**		
<37	29	9.4
≥37	279	90.6
**Height of mother**		
< 145 cm	12	3.9
≥145cm	296	96.1
**Weight gain during pregnancy**		
<10kg	118	38.3
≥10 kg	190	61.7
Mean ± SD	11.04 ± 4.15 kg	
**Weeks of gestation**		
<37 weeks of gestation	29	9.4
≥37 weeks of gestation	279	90.6
**Parity**		
Primiparous	161	52.3
Multiparous	142	46.1
Grand multiparous	5	1.6
**Birth Interval (n = 147)**		
Less than 2 years	12	8.2
More than 2 years	135	91.8
**Type of delivery**		
Cesarean Section	109	35.4
Normal Delivery	199	64.6
**Number of ANC visit**		
less than 4 times	26	8.4
4 or more times	282	91.6
**Intake of deworming tablet**		
Yes	128	41.6
No	180	58.4
**Hemoglobin level at first ANC visit**		
<11 gm/dl	38	12.3
≥11 gm/dl	270	87.4
Mean ± SD	12.27±1.28 gm/dl	
**Consumption of IFA tablet**		
Yes	300	97.4
No	8	2.6
**Number of IFA tablet intake**		
≥180 tablets	216	70.1
<180 tablets	92	29.9
Mean ± SD	153.25±43.06	
**Compliance with IFA supplementation**		
Non-compliance	69	22.4
Compliance	239	77.6
**Birth weight of baby**		
<2.5kg	47	15.3
≥2.5kg	261	84.7
Mean ± SD	2.96±0.59 kg	
**Knowledge of anemia and IFA intake**		
Inadequate knowledge	292	94.8
Adequate Knowledge	16	5.2
**Tobacco use during pregnancy**		
Yes	4	1.2
No	304	98.8
**Alcohol intake during pregnancy**		
Yes	4	1.2
No	304	98.8
**Food taboos**		
Yes	196	63.6
No	112	36.4

### Association of study variables with risk of low birth weight

Table 2 shows the factors associated with low birth weight. The result from regression analysis showed that the risk of low birth weight baby was more likely for mothers who belonged to Dalit ethnicity (AOR = 2.9, 95% CI = 1.2–7.1), mothers with IFA compliance (AOR = 2.1, 95% CI = 1.0–4.4), and Mothers who had four or more ANC (AOR = 2.6, 95%CI = 1.0–6.6).

**Table 2:** Factors associated with low birth weight.

Variables	Unadjusted OR	95% CI	P- value	Adjusted OR	95% CI	P- value
**Ethnicity**						
Dalit	3.6	1.5–8.5	0.003	2.9	1.2–7.1	0.017*
Non Dalit	1			1		
**IFA Compliance**						
Non Compliance	2.8	1.4–5.5	0.002	2.1	1.0–4.4	0.031*
Compliance	1			1		
**ANC visit**						
Less than four times	4.1	1.7–9.8	0.001	2.6	1.0–6.6	0.045*
Four or more times	1			1		

*Statistically significant (p< 0.05) at 95% CI

COR = Crude Odds Ratio, AOR = Adjusted Odds Ratio, Ref = Reference category

## Discussion

Our study found that about one in every seven children had a birth weight of less than 2500 gm. The three factors: (1) ethnicity, (2) Compliance with IFA, and (3) Maternal Antenatal care visits were significantly associated with birth weight of children. The prevalence of low birth weight (15.3%) was slightly lower than the study conducted in Dhulikhel hospital (21.6%) [34], Janakpur zonal hospital (21.5%) [35], Bharatpur teaching hospital (23.6%) [27] and higher than the National prevalence of (12.9%) [10] reported by Nepal Demographic Health Survey 2016. The prevalence of low birth weight was also higher in different regions or countries other than Nepal, such as in India (18%) [36], Ethiopia (17.3%) [37], Bangladesh (22%) [38], and in sub Saharan African countries (16.4%) [39]. But, the reported prevalence is higher than the study conducted in china (2.8%) [40], Jordan (13.8%) [29], and Malawi (12.1%) [41]. This difference could occur due to variation in study setting, socioeconomic and demographic characteristics of study participants.

Maternal age is considered as a key factor for the healthy outcome of pregnancy. This study reveals no statistical association between maternal age and low birth weight, which contradicts the study done in tertiary hospitals of Nepal that shows higher risk of delivering low birth weight babies by mothers of age less than 20 years [22, 30]. This might be because most of the participants were from age group 20–30 years, and the mean age was 25.75 years.

Our study showed that ethnicity significantly affects a baby’s birth weight. The mothers who belongs to Dalit ethnicity were nearly three times more likely to have low birth weight baby. This finding was consistent with another study conducted in Chitwan, which revealed that mothers belonging to Dalit and Madhesi ethnicity were more likely to have low birth weight baby [23]. Similarly, another study conducted in California showed African American women had 2 times more likely to have low birth weight compared to white women [42]. This might be due to the poor health care utilization, poor socio economic condition, and other social disparities faced by these ethnic groups in Nepal.

Our study revealed that mothers who did not attend four times or more antenatal services were 2.6 times higher change of low-birth-weight baby compared to those who attend four or more times. In support with this finding, another study conducted in Nepal using nationally representative survey data in 2011 showed that mothers who attend three or fewer ANC visits only were two times more likely to have low birth weight babies [31]. This finding was also consistent with the other studies conducted in sub Saharan Africa [39], Ethiopia [40], India [43], and the United States [44]. This might be due to during Antenatal visits, pregnant mothers are likely to receive health services such as nutrition counseling to improve dietary practices, encourage recommended weight gain during pregnancy, health assessment and receive nutrient supplement which is key to improving birth outcome [45].

Our study showed that 77.6% of mothers had compliance to iron-folic acid supplementation. A lower finding was seen in a study in Pokhara, Nepal that showed the compliance rate of 58% [32]. The studies in Karnataka and Senegal had similar findings, with 71% and 69% of the IFA compliance [46, 47]. However, only 18% of the compliance was seen in a study in Ethiopia [48]. These differences could be the result of different cut-offs for determining the IFA compliance in different countries. This study has considered 80% or above the intake of recommended dose as compliance, while different studies have used cut-off of 100 or the mean value.

Low iron intake causes the poor delivery of iron to the fetus, impair in proper hormonal and neuronal regulation of pregnancy and poor oxygenation of the fetus leading to poor growth and development [49]. Our study reflects that there is significant association of compliance to Iron folic acid supplementation with baby’s birth weight. Mothers who did not comply with iron-folic acid were two times more likely to have low birth weight babies. This finding is consistent with the findings of other studies [22, 50, 51], which revealed that IFA compliance has a positive association with baby’s birth weight. Every pregnant woman are recommended to take a total of 180 IFA tablets to reduce the risk of maternal anemia, and adverse outcomes including low birth weight. This is also supported by other studies’ findings, which showed higher odds of delivering low birth weight baby among non-compliant mothers [22, 50, 52].

Smoking and alcohol consumption negatively affects the fetus’s growth and development because of their chemical substances [53]. However, no significant association was seen with low birth weight in bivariate analysis. This is because of the very small portion of participants (only 8) who smoked and consumed alcohol. There may be possible social desirability bias because of social stigma.

This study has certain limitations that should be addressed in future research. The study was based on the health institutions limiting its generalizability in the community setting. Also, the other potential risk factors for low birth weight including micronutrient deficiencies among mothers and exposure to toxins like pesticides and indoor air pollution, were not evaluated, which may have affected the result.

## Conclusion

This study concluded that about one in seven children had low birth weight. The mothers who belong to Dalit ethnicity, who did not have antenatal visits as per recommended protocol (4 or more times) and mothers who did not comply with iron-folic acid supplementation were likelier to have low birth weight babies. This suggests that disadvantageous ethnic groups should be a focus in national preventive programs for the prevention of risk of delivering low birth weight baby. During ANC visits, priority should be given for providing nutrition education and motivating pregnant women towards IFA compliance focusing on complete ANC visits. Further studies such as case-control, cohort studies might help to bring forward the information on factors associated with the birth weight of children.

## Supporting information

S1 Data (SAV)

S1 File Questionnaire in English. (DOCX)
