# Supplementary material for: Prevalence of low birth weight and its associated factors: Hospital based cross sectional study in Nepal
Source: PLOS Glob Public Health. 2022 Nov 2;2(11):e0001220. doi: 10.1371/journal.pgph.0001220 (PMC10021178; doi:10.1371/journal.pgph.0001220)
Supplement: S1 File — (DOCX) [file pgph.0001220.s002.docx]

## S1 Questionnaire: Questionnaire in English

A001. Form Number:

| RESPONDENT IDENTIFICATION  Instruction: Please fill in the blank and circle the appropriate answer where appropriate. |
| --- |
| Date of Interview (dd/mm/yy):  Name of the Hospital: |
| A002. Interview Result Code  COMPLETED………………………………………..….1  REFUSED………………………………………….…....2  INTERVIEW PARTIALY COMPLETED………..…....3 |

| S. No | Question | Options | Skip pattern | |
| --- | --- | --- | --- | --- |
| **Socio-demographic information** | | | | |
| 01 | How old are you?(in completed years) | ………………… |  | |
| 02 | What is your caste/ethnicity? | 1. Dalit 1 2. Janajati 2 3. Madhesi 3 4. Muslim 4 5. Brahmin/chhetri 5 6. Others (specify) 99 |  | |
| 03 | What is the highest grade you have completed? | 1. Illiterate 1 2. Literate (read and write) 2 3. Primary 3 4. Secondary 4 5. Bachelor and above 5 |  | |
| 04 | What is your occupation, which is what kind of work you mainly do? | 1. Agriculture 1 2. Service 2 3. Business 3 4. Labor work 4 5. Housewife 5 6. Others (specify) 99 |  | |
| 05 | What is your permanent place of residence? | ………………………… |  | |
| **Health service related information** | | | | |
| 06. | How many times did you take antenatal care visit during this pregnancy? | ………………number of times |  | |
| 07 | How many months pregnant were you when you first received antenatal care for this pregnancy? | …………….months |  | |
| 08 | Did you receive antenatal checkup in the following months during this pregnancy? | Yes No   1. 4 months . . . . . .1 2 2. 6 months . . . . . 1 2 3. 8 months . . . . . 1 2 4. 9 months . . . . . 1 2 |  | |
| 09. | During the pregnancy did you take deworming tablet? | 1. Yes 1 2. No 2 |  | |
| **Maternal factors** | | | | |
| **Obstetric information** | | | | |
| 10. | How many completed pregnancies did you have? | …………. | If only 1, skip to Qn. 12 | |
| 11. | What is the birth interval between the previous and youngest child? | ………………..years |  | |
| 12. | What was the type of delivery of the baby? | 1. Normal Vaginal delivery 1 2. Caessarian section 2 |  | |
| 13. | When you got pregnant, did you want to get pregnant at that time? | 1. Yes 1 2. No 2 | If Yes, go to Qn 15 | |
| 14. | Did you want to have baby later or did you not want any more children? | 1. Later 1 2. No more child 2 |  | |
| 15. | Do you have history of abortion? | 1. Yes 1 2. No 2 |  | |
| **Behavioural information** | | | | |
| 16. | Did you smoke cigarettes during this pregnancy? | 1. Yes 1 2. No 2 | If No, go to Qn.19 | |
| 17. | During this pregnancy, did you stop smoking? | 1. Yes 1 2. No 2 |  | |
| 18. | At what months of pregnancy, did you stop smoking or using the tobacco products? | …………………. months |  | |
| 19. | On average, how many cigarettes did you smoke each day/week during this pregnancy?  (IF LESS THAN DAILY, RECORD WEEKLY) | …………………… per day/week |  | |
| 20. | During this pregnancy, have you consumed an alcoholic drink such as beer, wine, spirits, fermented cider or [jaad, raksi, tungba? | 1. Yes 1 2. No 2 | If No, go to Qn. 23 | |
| 21. | During this pregnancy, did you stop consuming alcoholic drinks? | 1. Yes 1 2. No 2 |  | |
| 22. | At what months of pregnancy, did you stop consuming alcoholic drinks? | ………..months |  | |
| 23. | How frequently have you had at least one alcoholic drink during this pregnancy? | 1. Daily 1 2. 5–6 days per week 2 3. 1–4 days per week 3 4. 1–3 days per month 4 5. Less than once a month 5 |  | |
| **Dietary information** | | | | |
| 24. | What was your meal pattern during this pregnancy? | 1. Less than that of before pregnancy 1 2. Equal to that of before pregnancy 2 3. More than that of before pregnancy 3 |  |  |
| 25. | What are the food and drink items that you did not consume during pregnancy due to prohibition/restriction and what was the reason behind? | List of food items Reason  List of drink items Reason |  |  |
| **Knowledge on anemia and IFAS** | | | |  |
| 26. | Have you heard of the illness Anemia? | 1. Yes 1 2. No 2 | If No, go to Q n. 30 |  |
| 27. | What do you think causes anemia during pregnancy? | a. Don’t know 0  b. lack of iron/folic acid in the body 1  c. lack of iron rich diet 2  d. increase in the blood volume 3  e. others (specify) 99 |  |  |
| 28. | What do you think are the signs and symptoms of anemia? | a. Don’t know 0  b. Feels weak 1  c. Looks pale 2  d. Headaches 3  e. Dizziness 4  f. Tiredness and easily fatigued 5  g. Others (specify) 99 |  |  |
| 29. | What do you think may be the consequences of anemia during pregnancy? | a. Don’t know 0  b. Premature birth 1  c. Low birth weight of baby 2  d. birth defects 3  e. maternal death 4  f. neonatal death 5  g. others (specify) 99 |  |  |
| 30. | What do you think are the methods of Anemia prevention during pregnancy? | a. Don’t know 0  b. Intake of IFA tablet 1  c. Intake of iron rich diet 2  d. Others (specify) 3 |  |  |
| 31. | Have you heard of IFA drug?  probe with pinkish/reddish coloured tablet and show the IFA pill samples) | 1. Yes 1 2. No 2 | If No, go to Qn.35 |  |
| 32. | What do you think are the benefits of this drug? | a. Don’t know 0  b. Prevents anemia among pregnant women 1  c. Protects mother from sicknesses 2  d. Gives mother strength during delivery 3  e. Increases amount of blood 4  f. Makes foetus grow healthy and strong 5  g. Others (Specify) 99 |  |  |
| 33. | What do you think are the risks of taking IFA drug? | a. No risks 1  a. Harmful for fetal growth 2  b. Causes big weight of baby 3  c.Results in complicated delivery 4  d.Others (specify) 99 |  |  |
| 34. | How often should IFAS be taken? | a. Don’t know 0  b. Once every week 1  c.Once daily 2  d.Others (specify) 99 |  |  |
| 35. | How long should you take IFAS tablet during pregnancy? | a. Don’t know 0  d.Throughout pregnancy after first trimester 1  e.Others (specify) 99 |  |  |
| **Information on iron folic acid supplementation** | | | |  |
| 36. | During this pregnancy, were you given or did you buy any IFA tablets?  (probe with pinkish/reddish coloured tablet and show the IFA pill samples) | 1. Yes 1 2. No 2 | If No, go to Qn. 45 |  |
| 37. | Did you consume folic acid pill alone without iron content?  (probe with white/brown coloured tablet and show the folic acid pill samples) | 1. Yes 1 2. No 2 | If No, go to Qn. 39 |  |
| 38. | How many months pregnant were you when you started taking folic acid pill? | …………………months |  |  |
| 39. | How many months pregnant were you when you started taking IFA pills? | ………………months |  |  |
| 40. | Did you receive IFA related counseling by health workers? | 1. Yes 1 2. No 2 |  |  |
| 41. | Did you have Family support for IFA supplementation intake? | 1. Yes 1 2. No 2 |  |  |
| 42. | Did you buy IFA tablet or were you given free of cost? | 1. Free 1 2. Paid 2 |  |  |
| 43. | Did you skip the IFA dose during this pregnancy? | 1. Yes 1 2. No 2 |  |  |
| 44. | Reasons for skipping the IFA dose | 1. Forgetfulness 1 2. Taste 2 3. Side effect 3 4. Travel 4 5. Others (specify) 99 |  |  |
| 45. | How many IFA pills did you take during your pregnancy?  (Probe with how many tablets per month/ how many tablets did she not consume) | …………………… |  |  |
| **Information from ANC card and Maternity Register** | | | |  |
| 46. | Hemoglobin level at recorded at each ANC visit | …………… gm/dl |  |  |
| 47. | Height of mother | ………………..cm |  |  |
| 48. | Weight of mother recorded at first ANC visit | …………….kg |  |  |
| 49. | Weight of mother recorded at term | …………………kg |  |  |
| 50. | Gestational age at birth | 1. <37 weeks 1 2. >=37 weeks 2 |  |  |
| 51. | Weight gain during pregnancy | …………….kg |  |  |
| 52. | Birth weight of baby | ……………….kg |  |  |
